# Network characterization of the Entangled Model for sustainability indicators. Analysis of the network properties for scenarios

**DOI:** 10.1371/journal.pone.0208718

**Published:** 2018-12-17

**Authors:** Pável Vázquez, Jesús A. del Río, Karla G. Cedano, Jiska van Dijk, Henrik Jeldtoft Jensen

**Affiliations:** 1 Instituto de Energías Renovables, Universidad Nacional Autónoma de México, Privada Xochicalco S/N Col. Centro, Temixco Morelos, Mexico; 2 Terrestrial department, Norsk institutt for naturforskning, Torgarden, Trondheim, Norway; 3 Department of Mathematics and Centre for Complexity Science, Imperial College London, South Kensington Campus, London, United Kingdom; 4 Centro de Ciencias de la Complejidad, Universidad Nacional Autónoma de México, Cd. Universitaria, Delegación Coyoacón, Ciudad de México, Mexico; 5 Institute of Innovative Research, Tokyo Institute of Technology, Nagatsuta-cho, Yokohama, Japan; Instituto Nacional de Medicina Genomica, MEXICO

## Abstract

Policy-makers require strategies to select a set of sustainability indicators that are useful for monitoring sustainability. For this reason, we have developed a model where sustainability indicators compete for the attention of society. This model has shown to have steady situations where a set of sustainability indicators are stable. To understand the role of the network configuration, in this paper we analyze the network properties of the Entangled Sustainability model. We have used the degree distribution, the clustering coefficient, and the interaction strength distribution as main measures. We also analyze the network properties for scenarios compared against randomly generated scenarios. We found that the stable situations show different characteristics from the unstable transitions present in the model. We also found that the complex emergent feature of sustainability shown in the model is an attribute of the scenarios, however, the randomly generated scenarios do not present the same network properties.

## Entangled sustainability

Sustainable development is a new vision towards problems that involve human needs. The WCED [1] defines it as the development that “seeks to meet the needs and aspirations of the present generations without compromising the ability to meet those of the future”. Growing concerns about the endurance of future generations have led to an increased interest in achieving sustainable development. For instance, the UN sustainable development agenda [2] has adopted a set of future goals for countries to end poverty, to protect the planet, and to ensure prosperity. Each goal has specific targets to be achieved, and concrete actions are proposed to be done, policy-makers require tools to find pathways towards sustainable development. These pathways are commonly studied in the environmental and social sciences, and the inclusion of complexity science and systems thinking has been extensively studied [3–6]. The understanding of the dynamics between humans and their surroundings, using a cybernetic approach, has gained an important popularity and computational tools have been widely proposed, some examples are in references [7–19]. In that matter the merge of disciplines and transdisciplinary research has created a new understanding through a cybernetics approach that explores the structure and dynamics of systems. In the case of sustainability, the cybernetic approach studies the dynamics between humans and their surroundings, offering new ways of how sustainability can be achieved.

Following a cybernetic approach, we have proposed the Entangled Sustainability model [20] for the identification and selection of a F relevant sustainability indicators that represent pathway towards sustainability. Indicators are widely used by researchers and policy-makers alike to design and promote policies, but there are many sustainability indicators [21] and the resources needed to follow them are usually limited. Therefore strategies to select a set of indicators are needed. There has been models [22–25] that combine a participatory and a cybernetic approach. The Entangled Sustainability model is a computational model with participatory components, the model identifiesprioritizes sustainable indicators within a municipalityregion to enable the municipalityregion to adequately assess progress towards sustainability.

The Entangled Sustainability model identifies a set of sustainability indicators that suitably represent a human system. In the model, sustainability indicators co-exist as a self-organizing system obeying defined rules, due to this we have used the term entanglement for the model as well as reference to the framework it is based [26]. The entangled characteristic has been previously studied [27]. The indicators co-evolve in a static network, showing the emergence of transitions between metastable and unstable situations that are the result of the individual mechanisms of interaction. In this paper, we characterize the network properties of the Entangled Sustainability model for the metastable and unstable situations present in the system. As the model can consider different specific scenarios, we compare the results for well-defined scenarios against random scenarios (see Appendix D in S1 File).

Here, we will detail a method to create specific scenarios, followed by a characterization of the network for different situations and scenarios. In addition, we will issue conclusions from the characteristics of these networks.

## The Entangled Sustainability model

Sustainability is a challenging concept to define, but it is commonly understood [28] that the main objective for sustainability is to provide to everybody everywhere and at any time the opportunity to lead a dignified life in his or her respective society. Sustainability has been defined [29] as a transition that “should be able to meet the needs of a much larger but stabilizing human population, to sustain the life support systems of the planet, and to substantially reduce hunger and poverty”, as well as increase justice and equity [30]. This means that sustainability should have, in theory, the capacity of any system or process to maintain itself indefinitely. It has also been proposed [29] that sustainability requires “significant advances in basic knowledge, in social capacity and technological capabilities to use it, and political will to turn this …into action”. In this report it is clear that technology, social organization and political action, must have an important role for sustainability. In reference [31] it was proposed that it is important “to understand the fundamental character of interactions between nature and society” this understanding proposes “societ’s capacity to guide those interactions along more sustainable trajectories.” Today’s study of sustainability is specially done by sustainability science [32–34], and integrated, place-based science.

**Fig 1:**
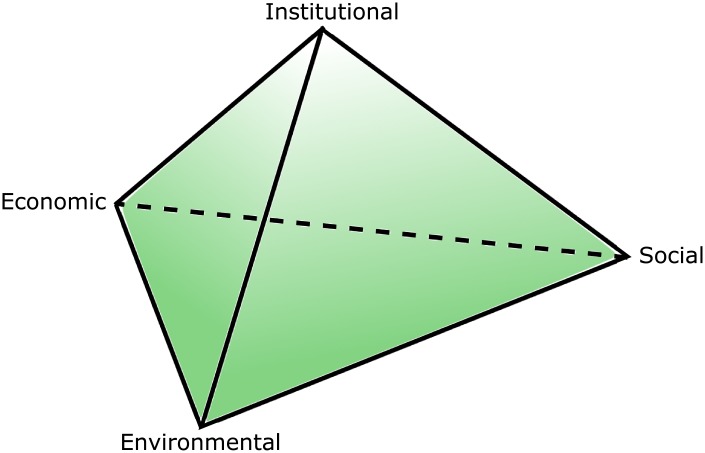
Four dimensions of sustainability. The economy refers the production and consumption of non-environmental and environmental goods and services together with the supply of money. The environmental dimension is the natural environment, such as water sources, biological life, forests, beaches, parks, etc. The social dimension concerns to the population living together, it refers to the equity, the diversity, the social cohesion, etc. Finally, the institutional dimension refers to organizations or other formal social structures, these organizations may be governmental agencies, NGOs, universities, sports clubs, families, etc.; but also this dimension includes social norms, principles, rules and decision-making procedures.

For conceptual and computational relevance, we have for our Entangled Sustainability model, used the Agenda 21 [35] conceptual understanding of sustainability as having four dimensions (see Fig 1),: social, economic, environmental and institutional. This definition allows the indicators to be seen as agents in a four-dimensional space, this means that each indicator can be described by a four-dimensional vector, also called agent *α*, so:

$$\begin{array}{c} I^{\alpha }=\left(Environmental,Economic,Social,Institutional\right). \end{array}$$

Each one of the vector’s entry can take four possible integer values in the range [0, 3]; these are the affinities of an indicator to each one of the dimensions of sustainability. Therefore 256 total indicators are possible. For example, an indicator described by the vector (3, 0, 0, 0) represents the economic dimension only, on the other hand, the indicator (2, 2, 2, 2) represents equally all dimensions.

In the Entangled Sustainability model indicators are referee to agents in a weighted network, so an indicator *I*^*α*^ is coupled with another indicator *I*^*β*^ with a value *J*(*α*, *β*) ≠ *J*(*β*, *α*). Each coupling can take values between [−*c*, *c*] and self-interaction is considered neutral (null).

The interaction between two indicators is the context of the model that we have called the scenarios. The indicators interaction is obtained from a 4 × 4 matrix called *J*^0^, shown in Eq (1) it creates the relations in the dimensions level. We propose the *J*^0^ interaction matrix to be flexible enough to simulate different scenarios, and it is composed as follows:

$$\begin{array}{cc} J^{0}= & \begin{array}{cc} & \begin{array}{cccc} En & Ec & So & In \end{array}\\ \begin{array}{c} En\\ Ec\\ So\\ In \end{array} & \left(\begin{array}{cccc} 0 & En\to Ec & En\to So & En\to In\\ Ec\to En & 0 & Ec\to So & Ec\to In\\ So\to En & So\to Ec & 0 & So\to In\\ In\to En & In\to Ec & In\to So & 0 \end{array}\right) \end{array} \end{array}$$ (1)

*J*^0^ relates dimensions, for instance the Environmental(*En*) with the Economic(*Ec*) as *En* → *Ec* or the Social(*So*) with the Institutional(*In*) as *So* → *In*. These values can be positive, negative or neutral. This way, the *J*^0^ matrix represents a scenario.

The indicator interactions are then created from the *J*^0^ matrix as described in Eq (2).

$$\begin{array}{c} J\left(\alpha ,\beta \right)=\sum_{i=1}^{L}\sum_{j=1}^{L}I_{i}^{\alpha }J_{ij}^{0}I_{j}^{\beta } \end{array}$$ (2)

Eq (2) creates another interaction matrix called the *J* matrix, which is the indicators interaction. A similar thermodynamic framework of irreversible thermodynamics has been previously studied [36].

**Fig 2:**
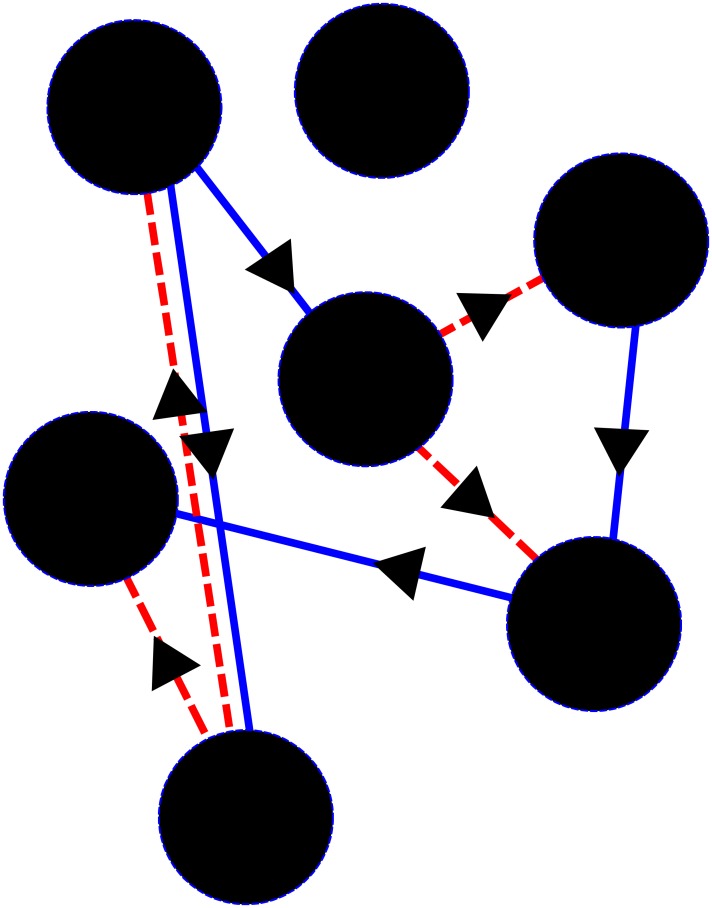
Example of a two-dimensional case of the network. Positive interactions are presented in blue. Meanwhile, a negative interaction is shown in red. Arrows indicate the origin of the interaction; an inward arrow means that the indicator receives fortitude and an outward arrow means that the indicator give fortitude.

The network of the model is exemplified in Fig 2, it is composed by the agents that represent indicators and the links between the indicators.

Indicators have an intrinsic propriety called the fortitude *F*^*α*^(*t*) of an indicator *α* at a time *t*. The fortitude represents the importance of an indicator in the system.

At time *t* + 1 each indicator has a chance to gain or lose fortitude given by a probability determined by the weight function shown in Eq (3).

$$\begin{array}{c} H\left(\alpha ,t\right)=a_{1}\frac{\sum_{\beta =1}^{N(t)}J\left(\alpha ,\beta \right)}{\sum_{\beta =1}^{N(t)}C\left(\alpha ,\beta \right)}-a_{2}\sum_{\beta =1}^{N(t)}C\left(\alpha ,\beta \right)-a_{3}\frac{N(t)}{R(t)} \end{array}$$ (3)

The first term of Eq (3) is the total sum of values in *J* of an indicator *α* with all of the other indicators *β* with *F*^*β*^(*t*) ≠ 0. The sum of values of *J* is then normalized by the total competition in the indicator space *α* with all the other indicators. The competition is an exponential function defined by:

$$\begin{array}{c} C\left(\alpha ,\beta \right)=\exp[\frac{\left(-1/4\right)\Delta I_{i}^{\alpha \beta }}{\xi }]\; \end{array}$$ (4)

with $\Delta I_{i}^{\alpha \beta }=\left|\sum_{i=1}^{4}\left(I_{i}^{\alpha }-I_{i}^{\beta }\right)\right|$, the distance between agents.

The second term of the weight function of Eq (3), *C*(*α*, *β*) is the competition, as given by Eq (4), two indicators affect each other negatively with a exponential decay.

The last term in Eq (3) is a growth regulation. The quantity *R*(*t*) is the number of available resources and *N*(*t*) number is the active number of indicators at time *t*. The sum of both quantities is kept constant, *R*(*t*) + *N*(*t*) = *const*.

Finally, to adjust the three terms in Eq (3) the coefficients *a*_1_, *a*_2_ and *a*_3_ are used.

As we have mentioned, each indicator has the capability of gaining or losing fortitude at each time step. This ability is related to the probability *P*_*g*_, which depends on the values of Eq (3).

$$\begin{array}{c} P_{g}=\frac{\exp[H(\alpha ,t)]}{1+\exp[H(\alpha ,t)]}. \end{array}$$ (5)

With probability *P*_*g*_ the indicator gains fortitude as in Eq 6, otherwise it will lose fortitude as in Eq (7).

$$\begin{array}{c} F^{\alpha }\left(t+1\right)=F^{\alpha }\left(t\right)(1+c_{g}\frac{J^{+}\left(\alpha \right)}{J_{Tot}\left(\alpha \right)}). \end{array}$$ (6)

$$\begin{array}{c} F^{\alpha }\left(t+1\right)=F^{\alpha }\left(t\right)(1-c_{p}\frac{J^{-}\left(\alpha \right)}{J_{Tot}\left(\alpha \right)}). \end{array}$$ (7)

The constants *c*_*g*_ and *c*_*p*_ are the gain and lose controls. *J*^+^(*α*) is the absolute sum of all positive interactions that the indicator *α* possess, meanwhile *J*^−^(*α*) is the absolute sum of all negative interactions. *J*_*Tot*_(*α*) is the indicator’s absolute if the total sum of the interactions that indicator.

In the case that *J*^+^(*α*) = 0 or *J*^−^(*α*) = 0, the *α* indicator does not participate in the dynamics. In that case, the absolute value of *J*^+^ or *J*^−^ falls below *J*_*min*_, so there is a probability of 1/2 to gain or loss of fortitude, given by:

$$\begin{array}{c} F^{\alpha }\left(t+1\right)=F^{\alpha }\left(t\right)(1+c_{g}\frac{J_{min}\left(\alpha \right)}{J_{Tot}\left(\alpha \right)}). \end{array}$$ (8)

$$\begin{array}{c} F^{\alpha }\left(t+1\right)=F^{\alpha }\left(t\right)(1-c_{p}\frac{J_{min}\left(\alpha \right)}{J_{Tot}\left(\alpha \right)}). \end{array}$$ (9)

Here *J*^+^(*α*) and *J*^−^(*α*) are substituted by *J*_*min*_, the lowest interaction value for such indicator.

Also, if an indicator has fortitude less than a threshold *U*_*k*_ the indicator is removed and *F*^*α*^(*t* + 1) = 0. This means that regarding the stakeholderś view on sustainability, this indicator is not significant enough. On the other hand, if an indicatorś fortitude is greater than a threshold *U*_*m*_ its fortitude will transfer a quantity *c*_*t*_ with a constant probability *P*_*t*_ to a neighbour within a radius *r*.

## Sustainability indicators and the Entangled Sustainability model

As we have explained, the model indicators are vectors in a four-dimensional space, that we have also referred to as agents. In this section, we will present how we will represent the vectors as measurable indicators. For that matter, we have proposed the use of a set of 96 indicators used by the Commission on Sustainable Development(CSD) [21]. According to the CSD, these core of 96 indicators covers most of the issues that are relevant for sustainable development, these indicators provide critical information and are easily calculated. Considering that the CSD have had a relevant role in sustainability and that the proposed guidelines have been extensively used, we have decided to use the CSD set of indicators for the Entangled Sustainability model.

Let’s remember that each indicator is a four-dimensional vector

$$\begin{array}{c} I^{\alpha }=\left(Environmental,Economic,Social,Institutional\right) \end{array}$$

with values in the range [0, 3]. Therefore, the association of the model and the CSD indicators was done by asking five experts to propose a value of affinity between each indicator on each one of the four dimensions of sustainability, see Fig A in S1 File. The resulting association is presented in S1 File.

The association showed to be fairly levelled in all dimensions. Also, indicators are associated close to vectors with intermediate values. Vector values with very high or very small values, for instance, (1, 0, 0, 0) or (3, 3, 3, 2), were rare, this result can show that the CSD set is intended to have indicators that are more extensive and able to cover a wider area of the sustainability.

All the CSD indicators were associated with one indicator from the model, but the number of indicators in the model is higher than the CSD set. Although not all agents represent indicators of the CSD, the agents can be modified slightly so that most agents can be associated to specific indicators that will be built in the necessary case.

### Scenario creation

The Entangled Sustainability model is intended to recreate regional situations that we have called the scenario. In the model, the *J*^0^ matrix is used to represent the interaction between sustainability dimensions and determine the scenario to be simulated, for that reason, the *J*^0^ matrix can be adjusted to represent a specific scenario (SS) adequately.

In [20] we have chosen the *J*^0^ values by directly asking experts in sustainability which value should the matrix take to represent the specific scenario. In this paper, we propose a different approach. We have created a survey (see Appendix B in S1 File) that we have used to ask different questions to local researchers and postgraduate students whose work is related to sustainability science.

We asked four questions for each sustainability dimension relation, see Eq (1). As we have 12 possible relations, then the survey consisted of 48 questions.

Thanks to the ability of collaboration with different institutions, three different scenarios have been used, Morelos and Jalisco regions in México and the region of Trondheim in Norway.

Researchers and postgraduate students were asked to give feedback on their level of agreement to statements that relate one dimension to another. We have used as reference the CSD guidelines [21] to relate different dimensions of sustainability in a region. For example, one of the questions used was: “Current availability of water has a positive effect on the economy of the region you live in.” Here we are positively relating an effect between the availability of water with the economy, i.e., the environmental dimension with the economic dimension. The possible answers were a range of agreement from the values (2, 1, 0, −1, −2) corresponding to the Completely agree, Agree, No opinion, Disagree, Completely disagree.

Then we obtained the mean of the same answer from different opinions, so we used these values to create the specific scenario for the *J*^0^ matrix using the Eq (2).

## Network of the model

In this paper we study networks emerging as a result of the dynamics of the model, we characterized the long stable situation in comparison with unstable situations and the network from scenarios created with the previous method along with randomly generated networks (see S1 File). We have also reported in [20] that specific values for a group of parameters produce the long stable situations; we will call these the standard values.

As we have explained, the Entangled Sustainability consists of sustainability indicators as nodes connected by links. It is important to mention that the model network is directed, i.e., each node has both in and out links.

**Fig 3:**
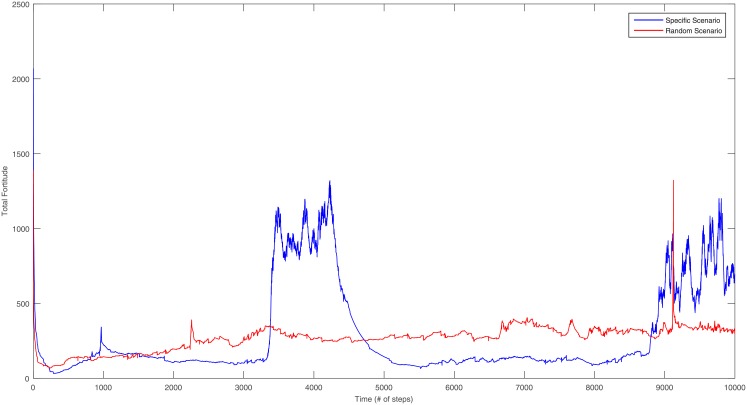
Comparison of the overall behavior of the entire system’s fortitude. We used the *J* ∈ [−100, 100], *P*_*t*_ = 0.3, *C*_*t*_ = 0.3 and *r* = 10.

We found that specific scenarios (SS) and randomly generated scenarios (RGS) present different qualitative result. For example, in Fig 3 two simulations are presented using for both cases the standard values. The use of an SS is shown in blue, here unstable situations are present with an increase and fluctuation of the total fortitude. Meanwhile, the case of an RGS is shown in red, where the unstable situations do not arise.

Using the information about the structure of the network at a time *t*, In the following subsections, we will explain three important measures of the networkś topology that we used: the degree distribution, the clustering coefficient, and the Interactions distribution.

### Degree distribution

The degree [37] is the number of links a node has with other nodes, measuring the degree of all nodes gives the probability *P*(*k*) that a randomly selected node has *k* links, which is then the degree distribution. A directed network like those in the model has both an in-degree and an out-degree distribution, these are the number of incoming and outgoing links. For simplicity, we did not distinguish the in-degree *k*_*i*_ and out-degree *k*_*o*_; hence we counted only the existence of a link between two nodes. If two nodes had both in and out link then the connection between nodes was counted only once.

Even though the in and out degree of a node is an important local quantity, the total degree distribution can determine the global characteristics of the network. For instance, the links in a randomly generated network are placed randomly, so most of the nodes have the same number of links to other nodes, i.e., they have the same degree. Meanwhile a Poison distribution [37] it is usually associated with a random network. But for most of the large complex networks, the degree distribution significantly deviates from a Poisson distribution. These networks are called scale-free and their degree distribution has a power-law tail of the form *P*(*k*) ∼ *k*^−*γ*^.

### Clustering coefficient

The clustering coefficient [37] is a measure of the degree to which nodes tend to cluster together. It is then the ratio between the number *E* of nodes that are present, divided by the *k* nodes and the maximum number of links *k*(*k* − 1)/2. For an indicator *α* the clustering is:

$$\begin{array}{c} C_{\alpha }=\frac{2E_{\alpha }}{k_{\alpha }\left(k_{\alpha }-1\right)}. \end{array}$$ (10)

With *k*_*α*_ the number of nodes of an indicator *α*. It is important to describe a network by measuring its tendency to form clusters. The clustering coefficient is then the number of closed links made by a group of at least three nodes, if this kind of triangle is created, then it is said that it is a cluster.

### Interaction strength distribution

Let us remember that if an indicator *α* with *F*(*t*) ≠ 0 then it will be connected to other indicators. The interaction strength distribution [38, 39] is measured by counting the number *N* of indicators that have a total sum of links *s*. So, all the links *J* of an indicator *α* are added to obtain the sum *S*, and then the interval of possible values of *S* is divided into boxes. Finally the number *N* of indicators that have that value *S* are counted.

## Results

As we have explained, the Entangled Sustainability model is composed of interconnected sustainability indicators, and the structure of the model’s network describes a specific sustainability scenario. For this reason, we have used the interaction matrix *J*^0^ to define the scenario.

In this section, we will distinguish two different networks: where a specific scenario(SS) *J*^0^ is previously defined (see the Scenario Creation section), and a random generated *J*^0^ network (RGS).

We will first characterize two different situations present in the model: the unstable situation and the metastable situation. In [20] we designed a test based on the Pareto principle [40] to identify stable situations, also known as the 80-20 rule, meaning that less than 20% of the population has more than 80% of the strength. The principle is asserted to appear in several different aspects of socioeconomic systems [41–43]. In our case, we use this property in order to define the Paretian set as the set of long lasting indicators that fulfil the Pareto principle.

In Fig 3 we present two simulations from two cases, the blue line corresponds to a single simulation from the SS for Morelos. In this example metastable and unstable situations are present, unstable situations are characterized by an increased and fluctuated total fortitude, and on the metastable situations, less fortitude movement is present. Contrary to the unstable situations, a Paretian set is constant and well defined during metastable situations. On the other hand, the case of a simulation using an RGS is shown in red. The total fortitude varies slightly, but no situations where the Paretian set is constant are present. This means that unlike the scenario network, the random scenario does not have a long-lasting Paretian set. We have observed that previous behavior in all simulations, so in the next sections we will present further differences and similarities of both cases, both in the SS and the RGS.

### Stability of simulations

In the Network of the model section, we have described the degree distribution, the interaction strength distribution, and the clustering coefficient as the main network measures that characterize the model network topology. To analyse if the SS and RGS are statistically different or similar, we have used different central tendency measures.

The degree distribution measures how connected the nodes are. To test if both distributions are different, in this case, we have used the *χ*-square test, using the null hypothesis that the specific distribution comes from the random sampling distribution. If the null hypothesis is rejected, then the degree distribution from both cases are different, if is not rejected both distributions are comparable. Using the same criteria, for the clustering coefficient, we have used Student T-test, as well we compared the two networks clustering with a correlation test. Meanwhile, as the interaction strength distribution provides a good qualitative insight, we will use it for that matter.

#### Degree distribution tests

First, we compared different simulations from the same SS and RGS, so we could test if different simulations are different. Finally, we compared SS and RGS between them. We have used the *χ*-squared test to determine if there is a significant difference on the frequencies of the degree distribution.

*χ*-square test was done by binning the number of interactions (*x* axis) into five fixed boxes and then the frequency of the links density (*y* axis) counted. Then, two frequencies are compared using *FE* as the expected frequency and *FO* as the observed frequency. The sum of these quantities over the five bins is the test statistic. The comparison is made with the *χ*-square test as follows:

$$\begin{array}{c} \chi^{2}=\sum_{i=1}^{5}\frac{{\left(FO_{i}-FE_{i}\right)}^{2}}{FE_{i}} \end{array}$$ (11)

Table 1, Table A in S1 File and Table B in S1 File show the *χ*-squared test of 3 SS. The test presents lower values than the critical value of 1.064, for 4 degrees of freedom and 90% of confidence. Thus with a 90% level of confidence the null hypothesis cannot be rejected, i.e., the SS are statistically equivalent.

**Table 1:** *χ*-square test of the scenario number 1.

Scenario 1.1 vs Scenario 1.2	1.0263
Scenario 1.1 vs Scenario 1.3	1.0118
Scenario 1.2 vs Scenario 1.3	1.0196

On the other hand, in Table 2, Table C in S1 File and Table D in S1 File we present the *χ*-squared test of 3 random generated scenarios. Using the same criteria as the SS, we show that with a 90% level of confidence the null hypothesis is rejected. Meaning that the SS simulations are statistically similar meanwhile the randomly generated scenarios are different.

**Table 2:** *χ*-square test of the random generated scenario number 1.

Random 1.1 vs Random 1.2	12.88
Random 1.1 vs Random 1.3	12.97
Random 1.2 vs Random 1.3	14.11

### Clustering coefficient tests

To test the clustering coefficient a two sample Student T-test was used along with the Pearson correlation coefficient. The Student T-test was performed as follows:

$$\begin{array}{c} t=\frac{{\bar{X}}_{1}-{\bar{X}}_{2}}{s_{p}\sqrt{2/n}} \end{array}$$ (12)

where

$$\begin{array}{c} s_{p}=\sqrt{\frac{s_{X_{1}}^{2}+s_{X_{2}}^{2}}{2}} \end{array}$$ (13)

Here ${\bar{X}}_{1}$, $s_{X_{1}}$ and ${\bar{X}}_{2}$, $s_{X_{2}}$ are the means and the standard deviations from two simulations to be compared. As we have compared the means of two different simulations, each one with a range of ten thousand data points, the degrees of freedom are considered infinite.

Same as the *χ*-Square test for the degree distribution, on Table 3, Table E in S1 File and Table F in S1 File for the SS and Table 4, Table G in S1 File and Table H in S1 File for RGS, we present the results of the Student T-test. Results show that the clustering coefficient on the SS simulations are similar and on RGS are not.

**Table 3:** Student T-test of the scenario number 1.

Scenario 1.1 vs Scenario 1.2	2.556
Scenario 1.1 vs Scenario 1.3	3.86
Scenario 1.2 vs Scenario 1.3	2.628

**Table 4:** Student T-test of the random generated scenario number 1.

Random 1.1 vs Random 1.2	41.41
Random 1.1 vs Random 1.3	18.79
Random 1.2 vs Random 1.3	22.24

The Pearson correlation coefficient was calculated using

$$\begin{array}{c} r=\frac{\sum_{i=1}^{n}\left(x_{i}-\bar{x}\right)\left(y_{i}-\bar{y}\right)}{\sqrt{\sum_{i=1}^{n}{\left(x_{i}-\bar{x}\right)}^{2}}\sqrt{\sum_{i=1}^{n}{\left(y_{i}-\bar{y}\right)}^{2}}} \end{array}$$ (14)

Here the *i* clustering value from the first scenario to be compared is denoted by *x*_*i*_ and the from the second scenario is *y*_*i*_. Meanwhile $\bar{x}$ and and $\bar{y}$ are the means. The number *n* denotes the number of samples, we used *n* = 10000. A comparison between an SS and a RGS using this coefficient is shown in Tables 5 and 6

**Table 5:** Correlation of the scenario number 1.

Scenario 1.1 vs Scenario 1.2	0.431
Scenario 1.1 vs Scenario 1.3	0.488
Scenario 1.2 vs Scenario 1.3	0.419

**Table 6:** Correlation of the random generated scenario number 1.

Random 1.1 vs Random 1.2	0.03628
Random 1.1 vs Random 1.3	0.02096
Random 1.2 vs Random 1.3	0.01465

The Pearson correlation coefficients show a higher correlation for the SS, meanwhile there is no correlation between RGS simulations.

### Metastable and unstable situations for specific scenarios

As we showed in the previous section, the SS simulations had similarities between them but compared RGS simulations do not present similarities. For that reason, for now on, we will present all results as the mean of five simulations but without changing any parameter or the specified SS.

To show the SS unique characteristics, here we will present the same measures and tests to understand the metastable and unstable situations in specific scenarios using the standard values. In Fig 3 we have presented in blue an example of the total fortitude for the Morelos scenario, during a period of ten thousand time steps we can notice that there are unstable situations where the Paretian set is not defined. Also, this instability is characterized by fluctuations of the fortitude.

**Fig 4:**
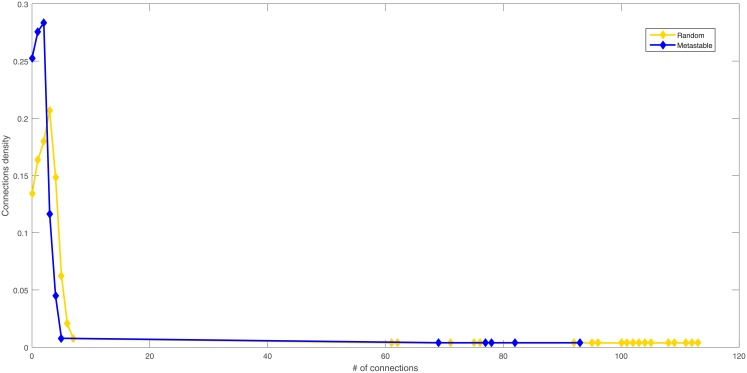
Degree distribution of the unstable and the metastable situations. Example using the Morelos scenario.

In Fig 4 the degree distribution of the unstable and metastable situations are exemplified using the Morelos scenario. The unstable situations are characterized by a small number of indicators that have few links and a fewer number of indicators with many links, on the other hand, the metastable situations also have a small number of indicators with many links but with more links. The metastable situations also have a small group of indicators with many links, but still, it is characterized by a higher number of indicators than the ones present in unstable situations.

The stable situation resembles a scale-free distribution with an adjusted *γ* = 2.55, meanwhile, the unstable situation has a lower *γ* = 2.32. This result resembles many real-world social networks [37].

Performing the *χ*-square test on the unstable against the metastable degree distribution we have obtained a value of 5.12, meaning that both distributions are different.

**Fig 5:**
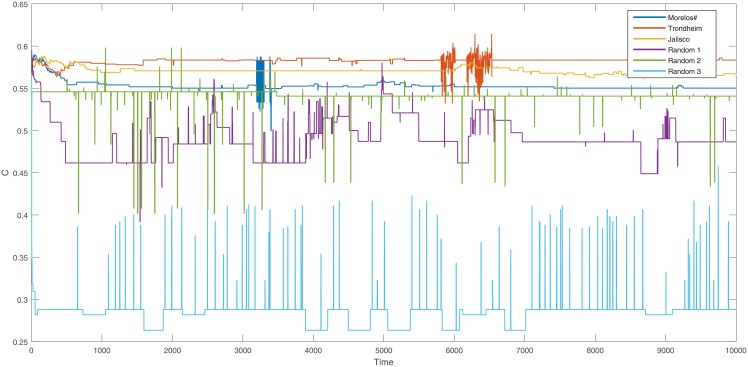
Clustering coefficient of different SS and RGS. Example using the Morelos scenario.

The clustering coefficient changes abruptly during the unstable situations, an example of the clustering coefficient for SS is presented in Fig 5. As the Student T-tests shows a value of 13.26 both cases are statistically different.

**Fig 6:**
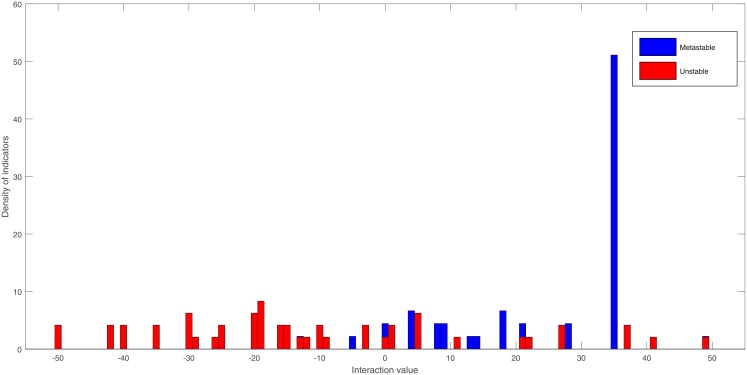
Interaction strength distribution of the unstable and the metastable situations. Example using the Morelos scenario.

The interaction strength distribution is also different in both situations. In the example shown in Fig 6 using the Morelos SS, the metastable situations have a mean connectivity of 22.5: meanwhile, the unstable situations have a mean of -10.4. Generally, the metastable situations show a higher number of indicators with positive connectivity, and the unstable situations present a wider distribution with negative connectivity.

### Scenarios

The two previous subsections showed important differences between simulation from the SS and the RGS. In the following subsection, we will show that the SS also produces a distinctive network for different scenarios. For that reason, we have compared SS using three different scenarios created from the survey method described in the Scenario creation section, and we will show the differences of these three SS along with other three different RGS.

To end, we will show the set of indicators from the Paretian set, that can sustain its composition during the stable situations, as a suggestion of the set of indicators to pursue a sustainable outcome.

#### Degree distribution

**Fig 7:**
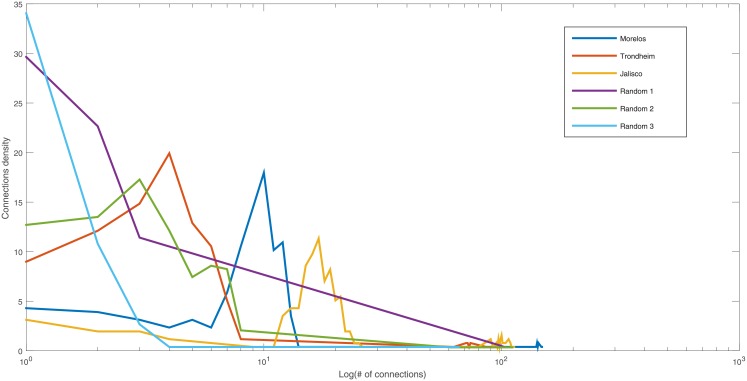
Degree distribution of different SS and RGS.

The degree distribution in a log plot is presented in Fig 7. The SS have similar degree distribution between them. If compared with the RGS, the SS has a peak of indicators with fewer links than the RGS. Also, the RGS resembles a power law distribution.

The *χ*-square test for the degree distribution of the three SS analyzed shown in Table 7, shows that the Morelos scenario is closer to the other two distributions. If we use the critical values for independence test (1,064 for 90% of confidence) only the Trondheim and Jalisco scenarios are statistically different.

**Table 7:** *χ*-square test for the degree distribution of scenarios.

Morelos vs Trondheim	0.707
Trondheim vs Jalisco	1.44
Morelos vs Jalisco	0.487

#### Clustering

The clustering coefficient presented in Fig 5 exhibits a similar behavior of the clustering as with total fortitude in Fig 3. In the SS cases the clustering is mainly stable, but similar to the fortitude, during the metastable situations, the clustering oscillates. This result adds another clear characteristic for the stable situation; these characteristics are: the Paretian set does not change, the total fortitude does not oscillate and here we show that also the clustering coefficient is stable.

It is also noticeable that the mean clustering coefficient of the SS and the RGS are different. Table 8 shows that the SS have a higher mean clustering coefficient than the RGS.

**Table 8:** Mean clustering coefficient for SS and RGS.

Morelos	0.5529
Trondheim	0.5822
Jalisco	0.5707
Random 1	0.4899
Random 2	0.5415
Random 3	0.2552

The values on Table 9 are the Student T-test for the clustering coefficient of the three SS analysed. The Morelos and Jalisco scenarios show the farthest distance. Using the critical value for independence test of 1.645 for a 90% confidence, only the Trondheim and Jalisco Scenarios are statistically similar.

**Table 9:** Student T-test for the clustering coefficient of scenarios.

Morelos vs Trondheim	3.92
Trondheim vs Jalisco	1.32
Morelos vs Jalisco	9.15

#### Interaction strength distribution

**Fig 8:**
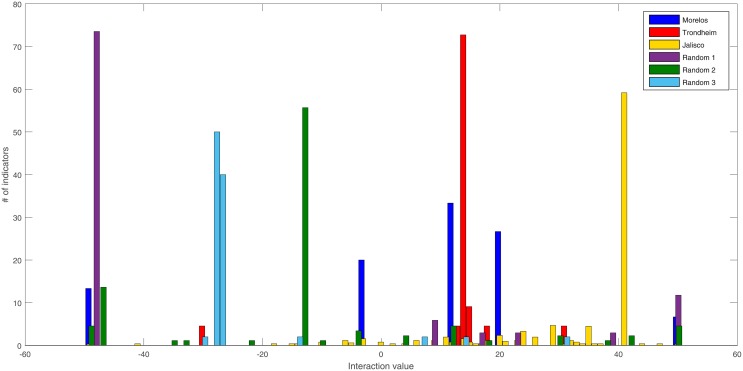
Interaction distribution of different SS and RGS.

We compared specific sustainability scenario and a random scenario. It is possible to notice in Fig 8 that both cases differ substantially. The SS it is characterized by fewer grouped indicators, and with positive values of total links, meanwhile the random case has mostly negative and dispersed indicators.

We also calculated the mean connectivity in Table 10. The SS are characterized by positive links, meanwhile, the three RGS mean of links is negative. This result shows that metastable situations only occur when positive interactions are present.

**Table 10:** Mean interaction strength distribution for SS and RGS.

Morelos	5.5
Trondheim	13.0
Jalisco	31.4
Random 1	-26.0
Random 2	-11.9
Random 3	-24.6

It is important to notice that the highest mean clustering coefficient and the most positive mean connectivity belongs to the Trondheim scenario, and in the degree distribution also Trondheim behaves as the scenario with most indicators with a higher number of links.

#### Indicators for the scenarios

The Entangled Sustainability model is proposed as a tool to select a set of indicators. As we have shown using specific scenarios, the model simulates a scenario with a network that is not similar to those of the random generated scenarios. The specific scenario network has shown stability, and also gives a well defined Paretian set of indicators as policy-makers require. Tables 11, 12 and 13 show the Paretian sets obtained for three specific scenarios created through the method described in the Scenario creation section.

**Table 11:** Indicators for the Morelos scenario.

Vector	Indicator name
0032	Number of intentional homicides per 100,000 population
0101	Share of imports from developing countries and from LDCs
0122	Number of internet users per 100 population
0133	Net enrollment rate in primary education
0231	Adult literacy rate, by sex
0322	Share of households without electricity or other modern energy services
0333	Vulnerable employment
1122	Percentage of total population living in coastal areas
1202	Arable and permanent cropland area
1333	Percent of population living below national poverty line
2103	Percent of forests damaged by defoliation
2122	Fragmentation of habitat
2200	Energy intensity of transport
2333	Net Official Development
3021	Land affected by desertification
3030	Proportion of fish stocks within safe biological limits

**Table 12:** Indicators for the Trondheim scenario.

Vector	Indicator name
0100	Share of imports from developing countries and from LDCs
0200	Domestic material consumption
0300	Current account deficit as percentage of GDP
0310	Mobil phones per 100 population
1100	Average tariff barriers imposed on exports from developing countries
1202	Arable and permanent cropland area
2210	Marine trophic index
2230	Remittances as percentage of GNI
2310	Area under organic farming
3300	Carbon dioxide emissions

**Table 13:** Indicators for the Jalisco scenario.

Vector	Indicator name
21	Suicide rate
31	Prevalence of tobacco use
111	Share of imports from developing countries and from LDCs
131	Share of women in wage employment in the non-agricultural sector
201	Investment share in GDP
211	Domestic material consumption
1011	Morbidity of major diseases such as HIV/AIDS, malaria, tuberculosis
1031	Proportion of urban population living in slums
1121	Percentage of total population living in coastal areas
1201	Arable and permanent cropland area
1221	Annual energy consumption per capita, total and by main user category
1231	Population growth rate
2131	Bathing water quality
2331	Net Official Development
3131	Generation of hazardous waste
3221	Land degradation

## Conclusions

Sustainability is the endurance of human systems over many generations, and through the social dynamics, the human systems adapt to metastable situations [3]. Here, we have developed a computational model that recreates this phenomenon to determine under which conditions metastability is present. In the Entangled Sustainability model, the complex interaction between indicators generate the emergence of an adapted Paretian set of indicators that may represent the sustainable situations.

We designed the *J*^0^ matrix on the Entangled Sustainability model to represent regional situations. This matrix condenses the interaction between sustainability dimensions. By using a survey of experts, we designed a methodology that provides the values of the *J*^0^ matrix. Thus, it is a methodology to create a specific *J*^0^ that represents a specific scenario. With this methodology, we could analyze the role of the scenarios on the stability of the model, this by comparing specific scenarios(SS) against randomly generated scenarios(RGS).

When simulating the same RGS multiple times, inconsistent network properties emerge, and the Paretian set cannot be determined. In comparison, the SS displays (during the stable situation) a well defined Paretian set that it is represented by a specific network configuration. This result implies that the network properties between the SS and the RGS are different, meanwhile, the network properties on the SS are similar.

We also characterized the metastable situation of the model so that we could understand its relevance. We compared the stable against the unstable situations using three different network measures, showing that the two networkś configurations are different and the properties of a stable Paretian set and resilient behavior are directly related to specific network characteristics. However, we emphasize that the stability of the Paretian set not imply that the indicators with more fortitude are stable as was explained in detail in [20].

The comparison between three different SS examples, from two different countries, displayed irregular results. The degree distribution and clustering coefficient of the Morelos 2 scenario showed similarities with other scenarios, mostly with the Trondheim and Jalisco scenarios. As so, the Jalisco and Trondheim scenarios show to be similar to the clustering coefficient.

Although these results are inconclusive, the comparisons of the SS display similarities between each other, as this property is not present in the RGS simulations. According to the values chosen for *J*^0^ we have shown that the model is capable of simulating different scenarios in regions. The stable situation has demonstrated to be characterized by a unique kind of network that can explain the resilient behavior of the Paretian set. The properties of the stable situation network also exhibit that the cooperation-competition dynamics create a network independent of the values of *J*^0^, but different from a random generated *J*^0^. The resulting set of indicators of specific scenario network, that we have previously reported as the Paretian set of indicators, establishes relevant indicators for this specific scenario. In conclusion, the results here presented show that the Entangled Sustainability model has a statistically equivalent stable network for each of the specific scenarios. These networks share centrality measurements. A different situation presents the randomly generated scenarios, which do not show stable network properties and show statistical differences in their centrality measurements. The Entangled Sustainability model is then suitable to identify a set of indicators that enable stakeholders to track pathways towards sustainable development in their localities.

## Supporting information

S1 File Appendix A. Fig A. Appendix B. Table A. Table B. Table C. Fig B. Appendix C. Table D. Table E. Table F. Table G. Table H. Table I. Table J. Table K. Table L. Table M. Table N. Table O. Appendix D.
(PDF)
