# Supplementary material for: Network characterization of the Entangled Model for sustainability indicators. Analysis of the network properties for scenarios
Source: PLoS One. 2018 Dec 17;13(12):e0208718. doi: 10.1371/journal.pone.0208718 (PMC6296531; doi:10.1371/journal.pone.0208718)
Supplement: S1 File — Appendix A. Fig A. Appendix B. Table A. Table B. Table C. Fig B. Appendix C. Table D. Table E. Table F. Table G. Table H. Table I. Table J. Table K. Table L. Table M. Table N. Table O. Appendix D. (PDF) [file pone.0208718.s001.pdf]

Appendix A

n order to associate the CSD indicators with the vectors of the model we have used the process detailed in Figure A. In this process each indicator is associated with a four-dimensional vector

$$I^{\alpha} = (Environmental, Economic, Social, Institutional)$$

that has values in the range $[0, 3]$. The association of the model and the CSD indicators was done by asking five experts to propose a value of affinity between each indicator on each one of the four dimensions of sustainability.

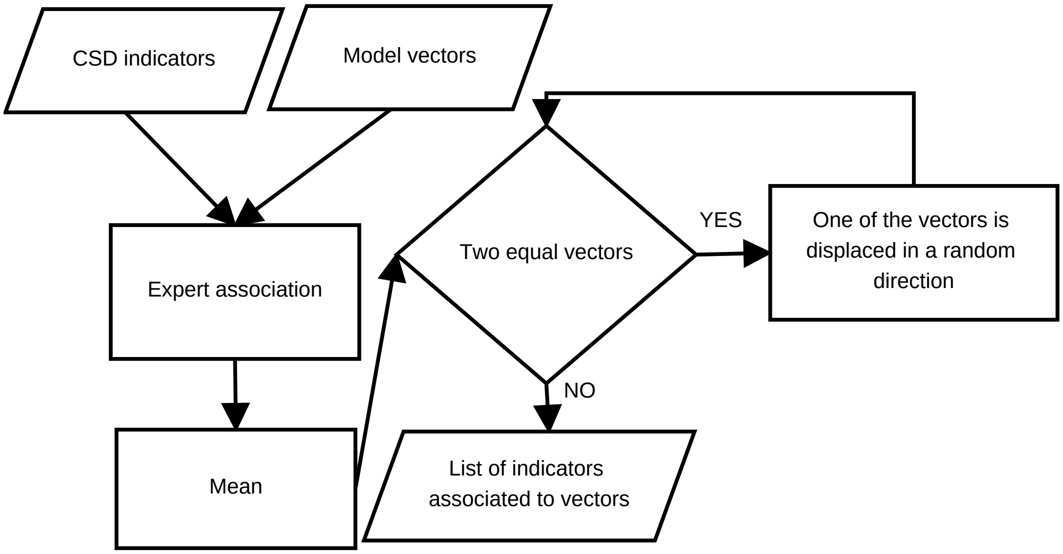

Figure A. CSD indicators association.

The result association was as show in the next table.

These indicators are the core indicators that the Commission on Sustainable Development(CSD) has proposed in order to cover most of the issues that are relevant for sustainable development, these indicators provide critical information and are easily calculated. Even though all the CSD indicators were associated with one indicator from the model, the number of indicators in the model is higher than the CSD set. Not all vectors represent indicators of the CSD, but the vectors can be modified slightly so that most agents can be associated to specific indicators that will be built in the necessary case.

The association was used in the Scenarios section in order to exemplify the differences between scenarios and it was also used to propose the Paretian set for each scenario.

Appendix B

We represent specific scenarios through defining a specific J^0 matrix constructed by a questionnaire applied to stakeholders. The responses to this survey were used as indicated in Figure B

Table A. Sustainability indicators association part 1.

10	Immunization against infectious childhood diseases
20	Gross intake into last year of primary education, by sex
21	Suicide rate
30	Total fertility rate
31	Prevalence of tobacco use
32	Number of intentional homicides per 100,000 population
33	Percent of population with access to primary health care facilities
102	Share of imports from developing countries and from LDCs
120	Number of internet users per 100 population
130	Contraceptive prevalence rate
131	Share of women in wage employment in the non-agricultural sector
133	Net enrollment rate in primary education
210	Domestic material consumption
212	Investment share in GDP
220	Assistance (ODA) given or received as a percentage of GNI
221	Ratio of local residents to tourists in major tourist regions
222	Human and economic loss due to natural disasters
230	Ratio of share in national income of highest to lowest quintile
231	Adult literacy rate, by sex
232	Gross savings
300	Current account deficit as percentage of GDP
310	Fixed telephone lines per 100 population
311	Dependency ratio
312	Debt to GNI ratio
313	Gross domestic product (GDP) per capita
320	Adjusted net savings

The possible answers to the questionnaire were in the range of agreement from the values $(2, 1, 0, -1, -2)$ corresponding to the Completely agree, Agree, No opinion, Disagree, Completely disagree. As there were four questions for the same J^0 matrix, then the four questions where averaged. Finally, each question with all the participants answers was averaged.

- Current permanent crops and monocultures have a positive effect on the economy[1] of the region you live in.
- Current use of fertilizers has a positive effect on the economy[1] of the region you live in.
- Current availability of water (used in the economy[1]) has a positive effect on the economy of the region you live in.
- Organic crops (crops that do not use synthetic products) have a positive effect on the economy[1] of the region you live in.
- Current air quality has a positive effect on human well-being in the region you live in.
- Current water quality has a positive effect on human well-being in the region you live in.
- Current abundance of species has a positive effect on human well-being in the region you live in.

Table B. Sustainability indicators association part 2.

321	Gross domestic expenditure subscribers per 100 population
322	Share of households without electricity or other modern energy services
323	Mortality rate under 5 years old
330	Employment-population ratio, by sex
331	Life long learning
333	Vulnerable employment
1020	Morbidity of major diseases such as HIV/AIDS, malaria, tubercolosis
1031	Proportion of urban population living in slums
1113	Average tariff barriers imposed on exports from developing countries and LDCs
1121	Percentage of total population living in coastal areas
1131	Adult secondary (tertiary) schooling attainment level, by sex
1132	Life expectancy at Birth
1200	Arable and permanent cropland area
1210	FDI inflows and outflows as percentage of GNI
1211	Material intensity of the economy
1212	Inflation
1220	Percentage of population using solid fuels for cooking
1221	Annual energy consumption per capita, total and by main user category
1231	Population growth rate
1301	Modal split of freight transport
1311	Tourism contribution to GDP
1320	Mobile cellular telephone subscribers per 100 population
1321	Labor productivity and unit labor cost
1332	Proportion of population below international poverty line
1333	Percent of population living below national poverty line
2011	Marine trophic index
2031	Proportion of population using an improved water source
2032	Proportion of population using improved sanitation facilities
2100	Proportion of total water resources used
2102	Proportion of marine area protected
2103	Percent of forests damaged by defoliation
2113	Proportion of terrestrial area protected, total and by ecological region

- Current abundance of green areas has a positive effect on human well-being in the region you live in.
- Normal fluctuating environmental conditions have a positive effect on the institutions[2] in the region you live in.
- Natural disasters have a positive effect on the institutions[2] in the region you live in.
- Current environmental laws have a positive effect on the reduction of air and water pollution in the region you live in.
- Current abundance of green areas has a positive effect on the institutions[1] in the region you live in.
- Current land use changes have a positive effect on the environment of the region you live in.
- Current industry in the region you live in has a positive effect on the air quality.

Table C. Sustainability indicators association part 3.

2122	Fragmentation of habitat
2131	Bathing water quality
2133	Percentage of population having paid bribes
2202	Energy intensity of transport
2203	Land use change
2211	Fertilizer use efficiency
2212	Generation of waste
2221	Modal split of passenger transportation
2222	Percentage of population living in hazard prone areas
2230	Remittances as percentage of GNI
2233	Use of agricultural pesticides
2310	Area under organic farming
2313	Share of renewable energy sources in total energy use
2333	Net Official Development
3000	Abundance of invasive alien species
3001	Abundance of selected key species
3003	Management effectiveness of protected areas
3011	Ambient concentration of air pollutants in urban areas
3013	Management of radioactive waste
3021	Land affected by desertification
3022	Proportion of fish stocks within safe biological limits
3100	Biochemical oxygen demand in water bodies
3101	Area of coral reef ecosystems and percentage live cover
3111	Proportion of land area covered by forests
3112	Area of selected key ecosystems
3121	Generation of hazardous waste
3122	Presence of fecal coliform in freshwater
3202	Water use intensity by economic activity
3211	Waste treatment and disposal
3212	Change in threat status of species
3213	Consumption of ozone depleting substances
3221	Land degradation
3223	Wastewater treatment
3232	Healthy life years expectancy
3233	Nutritional status of children
3311	Carbon dioxide emissions
3321	Emissions of greenhouse gases
3322	Area under sustainable forest management

- Current industry in the region you live in has a positive effect on the water quality.
- Current industry in the region you live in has a positive effect on deforestation.
- Job security in the region you live in is becoming uncertain.
- Current availability of consumer products has a positive effect on the economy[1] of households in the region you live in.
- Current availability of energy has a positive effect on the economy[1] of households in the region you live in.

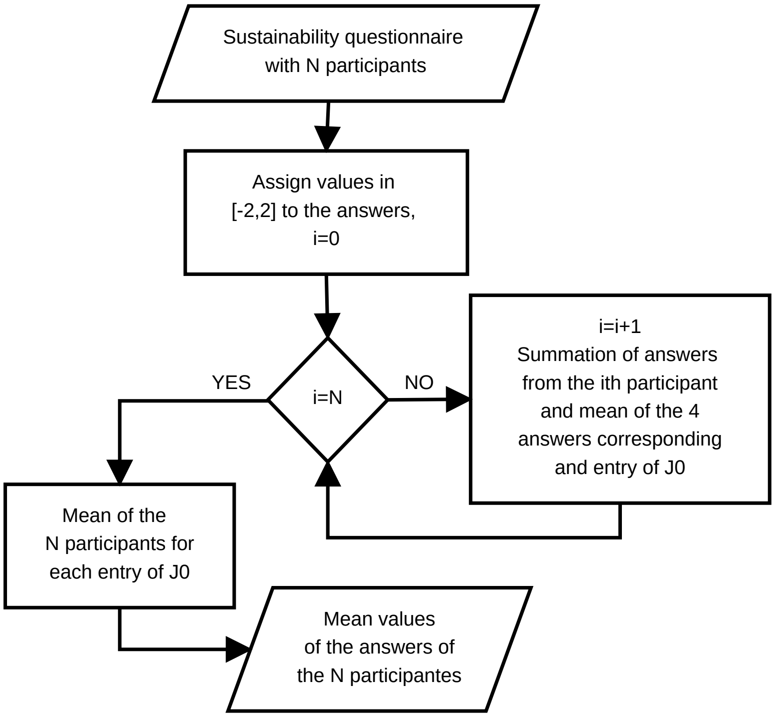

Figure B. Specific scenario creation.

- Current garbage recycling in households has a positive effect on the economy[1]in the region you live in.
- Current public and private spendings on education has a positive effect on the institutions[2] in the region you live in.
- Current labour productivity has a positive effect on the institutions[2] in the region you live in.
- Current public and private spendings on health has a positive effect on the institutions[2] in the region you live in.
- Current private and governmental funding and subsidies have a positive effect on the institutions[2] in the region you live in.
- Current commitment of citizens to recycling garbage has a positive effect on the environment in the region you live in.
- Current commitment of citizens to not pollute air and water has a positive effect on the environment in the region you live in.
- Current commitment of citizens to reduce water consumption has a positive effect on the environment in the region you live in.
- Current use of cars has a positive effect on the environment in the region you live in.
- Current amount of tourism has a positive effect on the economy[1] in the region you live in.

- Current amount of working hours has a positive effect on the economy[1] in the region you live in.
- The increase of working population has a positive effect on the economy[1]in the region you live in.
- Current labor efficiency has a positive effect on the economy[1] in the region you live in.
- Current corruption level has a positive effect on the institutions[2] in the region you live in.
- Current crime level has a positive effect on the institutions[2] in the region you live in.
- Current citizen implementation of the environmental laws has a positive effect on the institutions[2] in the region you live in.
- Current use of the social services by the citizens has a positive effect on the institutions[2] in the region you live in.
- Current engagement of institutions[2] in renewable energy issues has a positive effect on the environment in the region you live in.
- Current protection level of green areas has a positive effect on the environment in the region you live in.
- Current level of species protection has a positive effect on the environment in the region you live in.
- Current air and water pollution regulations have a positive effect on the environment in the region you live in.
- The promotion of tourism has a positive effect on the economy[1] in the region you live in.
- The promotion of sustainable economic practices has a positive effect on the economy[1] in the region you live in.
- The promotion of job creation has a positive effect on the economy[1] in the region you live in.
- Current business generation has a positive effect on the economy[1] in the region you live in.
- Job security has a positive effect on the society[3] in the region you live in.
- The promotion of culture has a positive effect on the society[3] in the region you live in.
- Current quality of the health services has a positive effect on the society[3] in the region you live in.
- Current quality of the education services has a positive effect on the society[3] in the region you live in.

[1] With the word 'economy' in the questionnaire, we mean the state of the region regarding the production and consumption of non-environmental and environmental goods and services together with the supply of money. It refers to both private and public organizations.

[2] With the word 'institutions' in the questionnaire, we mean organizations or other formal social structures that govern a field of action in the region we are analysing. These organizations may be governmental agencies, NGOs, universities, sports clubs, families, etc.; but also this dimension includes social norms, principles, rules and decision-making procedures. A positive effect on institutions can be for example an increased credibility, efficiency, security or empowerment.

[3] With 'society' we mean the population living together in the region we are analyzing. A positive effect on the society can be for example an increased cohesion, harmony and/or order.

This questionnaire was used with the methodology described in Scenario creation section in order to design the J^0 matrix and determine the scenario to be simulated.

Appendix C

Next we present the results of the statistical tests comparing the different scenarios. The test values on the three different tests consistently show lower values for the specific scenarios than for the random generated. For a 90% of confidence these values lead that the specific scenarios are statistically similar meanwhile the randomly generated scenarios are different.

Table D. χ-square test of the Trondheim scenario for the degree distribution.

Scenario 2.1 vs Scenario 2.2	1.0301
Scenario 2.1 vs Scenario 2.3	1.0322
Scenario 2.2 vs Scenario 2.3	1.0119

Table D shows the Chi-Square test using three different simulations for the same scenario. It shows that different simulations are statistically similar for the Trondheim specific scenario.

Table E. χ-square test of the Jalisco scenario for the degree distribution.

Scenario 3.1 vs Scenario 3.2	1.0128
Scenario 3.1 vs Scenario 3.3	1.0981
Scenario 3.2 vs Scenario 3.3	1.0121

Table E shows the Chi-Square test using three different simulations for the same scenario. It shows that different simulations are statistically similar for the Jalisco specific scenario.

Table F. χ-square test of the random generated scenario number 2 for the degree distribution.

Random 2.1 vs Random 2.2	13.56
Random 2.1 vs Random 2.3	11.89
Random 2.2 vs Random 2.3	13.08

Table F shows the Chi-Square test using three different simulations from a random generated scenario number 2. The result shows that the simulations are statistically different. Table G shows the Chi-Square test using three different simulations from the

Table G. χ-square test of the random generated scenario number 3 for the degree distribution.

Random 3.1 vs Random 3.2	16.51
Random 3.1 vs Random 3.3	13.23
Random 3.2 vs Random 3.3	10.47

Table H. Student T-test of the Trondheim scenario for the clustering coefficient.

Scenario 2.1 vs Scenario 2.2	3.825
Scenario 2.1 vs Scenario 2.3	3.819
Scenario 2.2 vs Scenario 2.3	4.0142

random generated scenario number 3. The result shows that the simulations are statistically different.

Table H shows the Student T-test using three different simulations from the Trondheim scenario. The result shows that the simulations are statistically similar.

Table I. Student T-test of the Jalisco scenario for the clustering coefficient.

Scenario 3.1 vs Scenario 3.2	2.692
Scenario 3.1 vs Scenario 3.3	3.0114
Scenario 3.2 vs Scenario 3.3	3.0122

Table I shows the Student T-test using three different simulations from the Jalisco scenario. The result shows that the simulations are statistically similar.

Table J. Student T-test of the random generated scenario number 2.

Random 2.1 vs Random 2.2	11.98
Random 2.1 vs Random 2.3	21.66
Random 2.2 vs Random 2.3	35.49

Table J shows the Student T-test using three different simulations from the random generated scenario number 2. The result shows that the simulations are statistically different. Table K shows the Student T-test using three different simulations from the

Table K. Student T-test of the random generated scenario number 3.

Random 3.1 vs Random 3.2	103.22
Random 3.1 vs Random 3.3	75.46
Random 3.2 vs Random 3.3	40.39

random generated scenario number 3. The result shows that the simulations are statistically different.

Table L. Correlation of the scenario number 2.

Scenario 2.1 vs Scenario 2.2	0.5021
Scenario 2.1 vs Scenario 2.3	0.585
Scenario 2.2 vs Scenario 2.3	0.593

Table L shows the Pearson correlation coefficient using three different simulations from the Trondheim scenario. The result shows that the simulations have a correlation.

Table M shows the Pearson correlation coefficient using three different simulations from the Jalisco scenario. The result shows that the simulations have a correlation.

Table M. Correlation of the scenario number 3.

Scenario 3.1 vs Scenario 3.2	0.391
Scenario 3.1 vs Scenario 3.3	0.386
Scenario 3.2 vs Scenario 3.3	0.389

Table N. Student T-test of the random generated scenario number 2.

Random 2.1 vs Random 2.2	0.00401
Random 2.1 vs Random 2.3	0.0178
Random 2.2 vs Random 2.3	0.00262

Table N shows the Pearson correlation coefficient using three different simulations from the random generated scenario number 2. The result shows that the simulations have no correlation.

Table O. Student T-test of the random generated scenario number 3.

Random 3.1 vs Random 3.2	0.8406
Random 3.1 vs Random 3.3	0.3039
Random 3.2 vs Random 3.3	0.4250

Table O shows the Pearson correlation coefficient using three different simulations from the random generated scenario number 3. The result shows that the simulations have no correlation.

These results show differences between simulations from different specific scenarios (SS) and random generated scenarios (RGS). The SS simulations showed to be statistically similar between them and RGS simulations does not. The results were used in the Stability of simulations section.

Appendix D

The random generated scenarios are built by assigning uniform random float number in the range [-2,-2] to the J0 matrix of Eq(1), following by the same procedure of creating the interaction matrix J as in Eq(2).
